# Vitreous humor analysis for the detection of xenobiotics in forensic toxicology: a review

**DOI:** 10.1007/s11419-015-0294-5

**Published:** 2015-10-28

**Authors:** Fabien Bévalot, Nathalie Cartiser, Charline Bottinelli, Laurent Fanton, Jérôme Guitton

**Affiliations:** Laboratoire LAT LUMTOX, 71 Avenue Rockefeller, 69003 Lyon, France; Institut de Médecine Légale, Université de Lyon, Université Claude Bernard Lyon 1, Faculté de Médecine Lyon Est, 8 Avenue Rockefeller, 69373 Lyon Cedex 08, France; Laboratoire de Toxicologie, ISPB-Faculté de Pharmacie, Université de Lyon, Université Claude Bernard Lyon 1, 8 Avenue Rockefeller, 69373 Lyon Cedex 08, France; Département de Médecine Légale, Hôpital Edouard-Herriot, Hospices Civils de Lyon, Place D’Arsonval, 69437 Lyon Cedex 03, France; CREATIS CNRS UMR 5220, INSERM U1044, Université de Lyon, Université Claude Bernard Lyon 1, INSA Lyon, 7 Avenue Jean Capelle, 69621 Villeurbanne Cedex, France; Laboratoire de Pharmacologie-Toxicologie, Centre Hospitalier Lyon-Sud, Hospices Civils de Lyon, 165 Chemin Grand Revoyet, 69495 Pierre Bénite Cedex, France

**Keywords:** Alternative matrices, Vitreous humor, Xenobiotics, Blood-retinal barrier, Postmortem redistribution

## Abstract

Vitreous humor (VH) is a gelatinous substance contained in the posterior chamber of the eye, playing a mechanical role in the eyeball. It has been the subject of numerous studies in various forensic applications, primarily for the assessment of postmortem interval and for postmortem chemical analysis. Since most of the xenobiotics present in the bloodstream are detected in VH after crossing the selective blood-retinal barrier, VH is an alternative matrix useful for forensic toxicology. VH analysis offers particular advantages over other biological matrices: it is less prone to postmortem redistribution, is easy to collect, has relatively few interfering compounds for the analytical process, and shows sample stability over time after death. The present study is an overview of VH physiology, drug transport and elimination. Collection, storage, analytical techniques and interpretation of results from qualitative and quantitative points of view are dealt with. The distribution of xenobiotics in VH samples is thus discussed and illustrated by a table reporting the concentrations of 106 drugs from more than 300 case reports. For this purpose, a survey was conducted of publications found in the MEDLINE database from 1969 through April 30, 2015.

## Introduction

Vitreous humor (VH), also known as the vitreous body, is a gelatinous substance contained in the posterior chamber of the eye, between the crystalline lens and the retina. It plays a mechanical role, keeping the retina in place and maintaining the spherical shape and tonus of the eyeball. There have been numerous studies of VH in various forensic applications. The first was to the assay of VH potassium, released during postmortem membrane degradation, as a means of estimating time of death [1]. Studies have found variable and sometimes contradictory results, depending on the authors, experimental conditions, analytic methods and statistical models [2–7]. Other means of achieving the same objective have been proposed: hypoxanthine assay isolated [8, 9] or associated to potassium [10, 11], amino acids [12] or creatinine assay [13], or VH absorbance assessment [14]. More recently, in a study using proton nuclear magnetic resonance (^1^H NMR) multivariate analysis of goat VH samples, Rosa et al. [15] recommended analyzing the global metabolite profile rather just than one or a few metabolites. A combined potassium and chlorine assay was reported for determining the immersion time of a body in cold water [16]. The other main applications of VH in forensics relate to postmortem biochemistry for screening or confirming preexistent pathology and determining cause of death (Table 1) [17–50].

**Table 1:** Postmortem diagnostic applications of vitreous humor

Application	Analysis	Reference(s)
Postmortem identification	DNA	[17]
Virology	Anti-HIV antibodies, proviral DNA	[18–24]
	Anti-adenovirus antibodies	[25]
Anaphylactic shock	Beta-tryptase	[26]
Sudden infant death	Multiple biochemical parameters Hypoxanthine	[27–29] [30]
Death from hypothermia	Catecholamines Amylase and isoamylase Glucose Ketone bodies	[31] [32] [33] [34]
Death from hypoxia	Hypoxanthine	[35]
Chronic excessive alcohol consumption	Zinc	[36, 37]
	CDT	[38, 39]
Alcoholic acidoketosis	Ketone bodies	[40, 41]
Intoxication by bleach	Sodium and chlorine ions	[42]
Brain damage assessment	Aminopeptidase	[43]
Self-induced water intoxication	Sodium	[44]
Endocrine disorder	Hormones	[45]
Glycemia	Glucose and/or lactates	[46–49]
Pregnancy	Chorionic gonadotropin	[50]

*CDT* carbohydrate-deficient transferrin

In forensic toxicology, VH has served as an alternative matrix for more than 50 years [51, 52]. Its lack of vascularization, anatomic remoteness from viscera, and relative protection by the eyeball render VH a useful alternative when blood cannot be sampled (exsanguinated or fragmentary cadaver) or in the case of suspected postmortem redistribution [53–56] or contamination by bacteria or chemicals (e.g., embalming) [57, 58]. As it is easy to sample, and because it can be used for immunological analysis of certain groups of chemical substances [59], VH has even been recommended for immunoenzymatic screening on the site where a victim was discovered [60]. While screening applications are acknowledged for a large number of compounds, the use of VH analysis for interpreting concentrations seems more limited.

The present literature review has two objectives. The first, by describing the physiology of VH and drug transport and elimination, is to suggest possible lines of research to improve our knowledge of forensic applications of this matrix. The second is to develop a practical tool for use at all levels of investigation using VH: sampling and sample storage, analytic techniques and interpretation of results. For this second objective, we conducted a MEDLINE search with “vitreous” as a keyword combined with “forensic sciences”, “toxicology”, “postmortem”, “post-mortem”, “autopsy” and/or “chromatography drug” (update, April 30, 2015). The research was restricted to the organic compounds most frequently encountered in forensic toxicology: medical drugs and narcotics. Ethanol was the first substance for which VH concentrations were interpreted [61]. The importance of VH quantification of ethanol has been widely studied and thoroughly reviewed by Kugelberg and Jones [62], and is therefore not dealt with in the present review.

## Physiology and pharmacokinetics

### Anatomy and composition of vitreous humor

**Fig. 1:**
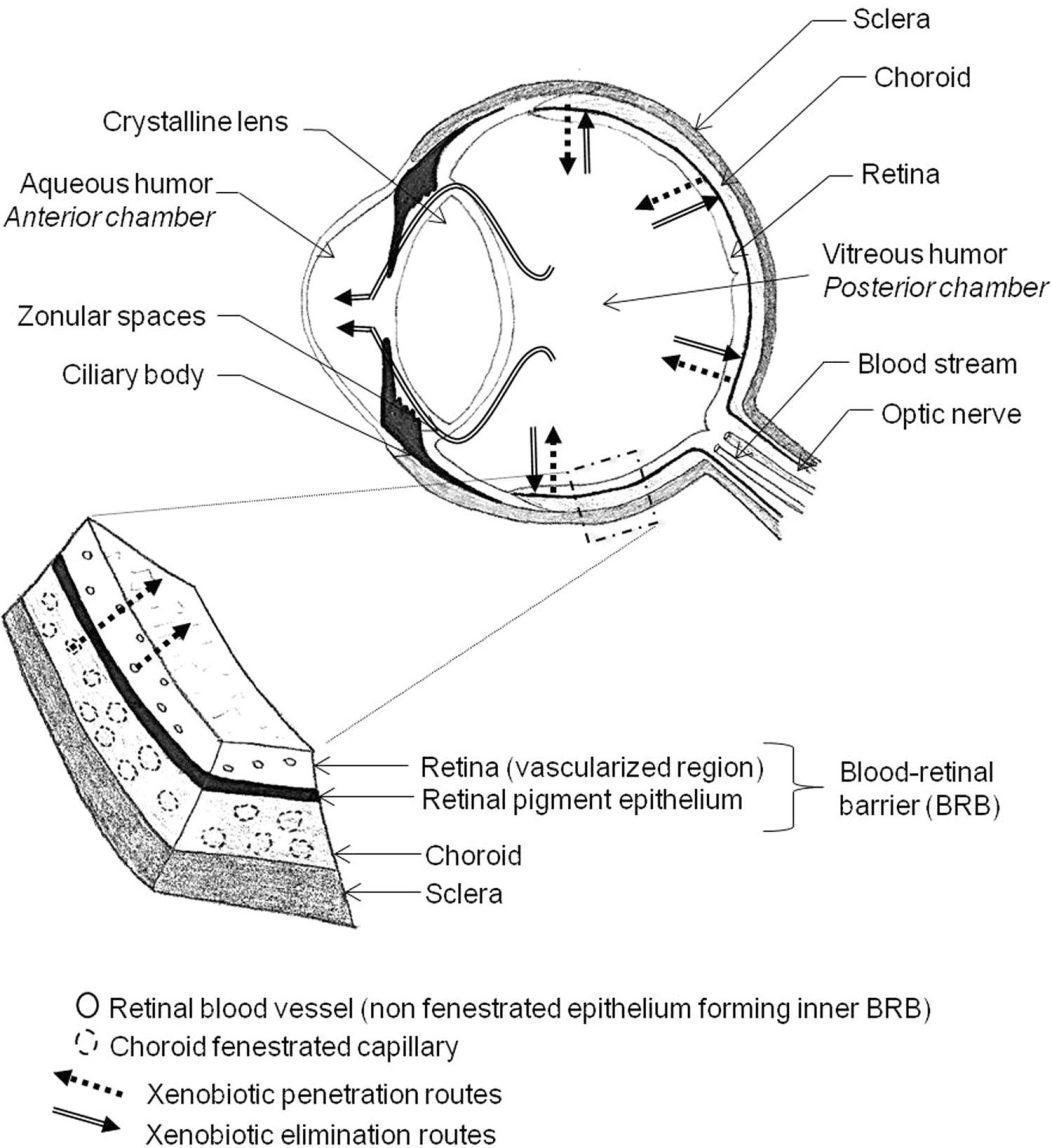
Anatomy of the eye and the blood-retinal barrier (adapted from [63])

The crystalline lens separates the anterior chamber of the eye, which contains a liquid (aqueous humor), from the posterior chamber, which contains VH (Fig. 1) [63]. The posterior chamber is bounded at the back, from inside to outside, by the retinal membrane, the choroid and the sclera, and at the front by the ciliary body and the crystalline lens. VH is highly hydrated tissue, with 98–99.7 % water content, and mean volume of 4 mL. Its gelatinous structure is due to fibrillar proteins: primarily collagen fibers associated with glycosaminoglycan carbohydrates (mainly hyaluronic acid). As many as 1205 proteins have been identified in the VH [64]. It also contains electrolytes (such as sodium, potassium, chlorine, lactate and ascorbate), carbohydrates such as glucose, and small amounts of proteins other than collagen, including opticin. VH is avascular and very poor in cells. Of the few cells, hyalocytes, or vitreous cells, are involved in synthesizing the constituents of VH and in the adaptive immune response that limits intraocular inflammation [65]. A change in composition with age leads to gradual liquefaction. In persons at the age of 4 years, liquid VH accounts for 20 % of the total vitreous volume, increasing to over 50 % by 80–90 years of age [66].

### The blood-retinal barrier

The blood-retinal barrier (BRB) is a selective barrier, like the blood-brain barrier [67]. It ensures the input required for retinal function and restricts that of possible pathogens (e.g., enzymes, anaphylatoxins) [68]. It actually comprises two barriers (Fig. 1). The first, comprising the retinal pigment epithelium (RPE) separating the retina from the choroid, is the outer BRB. RPE cells have the particularity of being bound together by intercellular junctions (*zonula adherens* and *zonula occludens*), forcing the intracellular transit of compounds. The second, which constitutes the non-fenestrated epithelium of the retinal blood vessels, is the inner BRB. The two barriers are not successive; rather, they are associated with the two retinal penetration pathways: choroid capillaries for the outer BRB and retinal capillaries for the inner BRB. Selectivity may be impaired by various pathologies, the most frequent of which are diabetic retinopathy and age-related macular degeneration [69].

### Xenobiotic exchange between blood and vitreous humor

In certain inflammatory or infectious ophthalmic pathologies, the posterior chamber is a drug target. Eyewashes and systemic treatments generally fail to achieve effective doses in VH; periocular and intra-vitreous injection is increasingly used for administration, although with a risk of infection. Compounds of forensic interest derive mainly from the systemic circulation, penetrating the VH from the retina via the BRB [70]. Two elimination routes from the VH have been described: a posterior pathway through the BRB in the opposite direction, and an anterior pathway by diffusion into the aqueous humor via the zonular spaces (Fig. 1), with elimination by the renewal of aqueous humor and uveal blood flow [71].

### Factors affecting xenobiotic penetration into the vitreous humor

Drug penetration into the retina depends on various factors, including plasma concentration, compound physicochemical and pharmacological properties, distribution volume, plasma protein binding and relative BRB permeability [70]. Drugs may diffuse passively or be actively transported across the barrier: in general, the higher the molecular weight and/or hydrophilicity, the more likely that passage across a membrane involves active transport [72]. Given that only non-bound drugs can cross biological membranes, the percentage of plasma protein binding is another factor determining diffusion. In a study of numerous compounds of forensic interest, Holmgren et al. [73] found significant correlation between blood/VH concentration ratios and percentage of plasma protein binding.

There are many transmembrane proteins expressed in the BRB that can act as transporters, playing a role in drug bioavailability in the posterior chamber. Two main types can be distinguished: efflux pumps, belonging to the ABC (ATP-binding cassette) transporter superfamily, and uptake pumps, belonging to the solute carrier (SLC) transporter superfamily. The main efflux transporters identified in the eye are multidrug resistance (MDR) transporters, including P-glycoprotein (P-gp or MDR1), multidrug resistance protein (MRP) and breast cancer resistance protein (BCRP). Unlike passive diffusion, active transport may be limited by saturation, if drug concentration exceeds transport capacity, and competition with other compounds or inhibition by certain specific substrates. Animal studies of concomitant administration of verapamil, a P-gp inhibitor, found longer VH elimination half-life for quinidine, whether administered intravitreously [74] or intravenously [75]. In forensic toxicology, such interactions may have a significant impact on the interpretation of VH concentration, especially as it affects the VH/blood concentration ratio.

There have been numerous studies of the VH pharmacokinetics of drugs used in ophthalmic therapy (e.g., antibiotics and anti-inflammatory agents), and of their transporters in particular. On the other hand, much less is known about compounds of general interest in forensic toxicology. The relative VH bioavailability of memantine was reported to be only 0.02 % after intravenous administration compared to intravitreous administration as reference; the concentration peaked at 29.68 ± 13.9 min, and the rapid elimination half-life (<2 h) argued for retinal elimination by active transport [76]. The research by Pitkänen et al. [77] into the effect of beta-blocker size and lipophilicity on both uptake and efflux permeation through the outer BRB is especially interesting. The most hydrophilic beta-blocker showed permeability coefficients that were seven- to eightfold lower than those for the most lipophilic beta-blockers (metoprolol, timolol and betaxolol). Atenolol uptake and efflux speeds were identical, whereas more lipophilic beta-blockers showed penetration faster than outflow. This permeation asymmetry in highly lipophilic beta-blockers may be due to an active transport component. Moreover, VH diffusion time was longer for lipophilic than for hydrophilic beta-blockers (permeation lag time for betaxolol = 107 min, versus 38.7 min for atenolol). Pitkänen et al. [77] suggested that this could be the consequence of drug binding to melanin: the outer BRB contains melanin, which is a molecular site for basic and lipophilic drug binding and interaction [78], influencing permeation.

The various transport mechanisms and the factors governing them are important for the understanding and description of the distribution of drugs from blood to VH. These factors seem to affect low-molecular-weight molecules: i.e., most compounds of forensic interest. Evidence of their exact impact on the interpretation of VH concentrations, however, is rarely documented in the forensic literature, except for plasma protein binding.

## Postmortem evolution of vitreous humor

VH tends to liquefy according to postmortem interval and local conditions. Postmortem evolution involves dehydration, which some authors have assessed in terms of increased creatinine concentration [56]. To our knowledge, there have been no studies of VH bioavailability relative to postmortem time. In our own experience, VH was sampled in 80 % of autopsies performed in the Forensic Medicine Institute of Lyon (France) between 2010 and 2013.

## Analysis of vitreous humour

### Sampling and storage conditions

VH is sampled by syringe, and aspiration should be slow, from the center of the eyeball, to avoid epithelial cells of the retina or iris. For the same reason, volume must be limited to 2 mL per eye, even though the total volume of VH is greater [79]. The volume withdrawn may be replaced by water or physiological saline in order to maintain the aspect of the eyeball [80]. Total versus micro-aliquot (50 µL) sampling procedures were compared in a rabbit model [81]. Micro-sampling seemed more reproducible for ion assay (calcium, chloride, potassium, sodium and phosphorus), but is too limited at present for forensic toxicology investigation.

Harper et al. [79], in a study of 51 paired VH and femoral blood samples, found that VH samples were less subject to bacterial contamination, which is an advantage in terms of sample and xenobiotic stability during storage; to enhance this advantage, the authors recommended sampling under aseptic conditions (for syringe and container) to avoid bacterial contamination.

Electrolyte and glucose samples have been reported to be lateralization-sensitive [82–85]. Rather than reflecting differential concentration between the two eyeballs, this may be a question of reproducibility related to the sampling procedure [81], of analytical problems due to the gelatinous consistency of VH [86], and of hematic contamination [87]. Bévalot et al. [88], in a series of 92 human autopsies, found the left/right VH differential to be non-significant for meprobamate. Findings were similar for 3,4-methylenedioxymethamphetamine (MDMA) [89], phenytoin [90], barbiturates [90] and cocaine [91]. However, for compounds such as digitalis-glycoside, which accumulates dramatically in the retina [92], sampling problems such as choroid and retinal cell aspiration may affect observed concentrations, and we recommend separate sampling of the two VH specimens, without pooling.

The container should be suitable for the small-sample volume: 5-mL tubes are preferred to the classical autopsy vials used for most samples in order to limit headspace gas volume and, thus, evaporation of volatile substances such as ethanol [93].

Although VH is generally considered unaffected by postmortem enzymatic and bacterial phenomena (few cells, protected from bacterial contamination, etc.), there have been several reports using stabilizers such as sodium fluoride (NaF) or potassium fluoride (KF) to block enzyme activity, causing neoformation or degradation of certain xenobiotics. Holmgren et al. [73] assessed the effect of KF on blood and VH concentration stability in 46 drugs. VH samples were divided in two, with KF added to one aliquot; all aliquots were conserved for 1 year at −20 °C. Among the 46 drugs, only zopiclone (*n* = 13) showed a significant reduction in mean concentration without the stabilizer (KF), from 0.15 to 0.03 µg/g. Moreover, 6-monoacetylmorphine (6-MAM) was exclusively detected in samples with KF (number of samples and concentration unknown).

Melo et al. [94] studied temperature effects on VH stability for benzodiazepines (lorazepam, estazolam, ketazolam and chlordiazepoxide). There was no significant degradation over 6 months in sub-zero storage (−20, −80 °C). Some benzodiazepines were relatively stable for a few weeks at +4 °C and +25 °C, whereas ketazolam degraded completely within 12 weeks at these temperatures. The stability of cocaine in ovine VH was studied by Rees et al. [95], with and without stabilizer (NaF), for 84 days at three temperatures: room temperature, +4 °C and −18 °C. At −18 °C, concentrations were stable (loss <15 %) for 84 days, both with and without NaF, and they were unstable at +4 °C, with a loss of 25 and 50 % by day 14, with and without NaF, respectively. The same team also studied the stability of 6-MAM [96]. The addition of 1.5 % NaF had a much clearer effect, limiting degradation to <10 % at −18 °C for 84 days, compared to 42 % by day 14 and 95 % by day 84 without a stabilizer. At +4 °C, likewise, degradation was <10 % on day 35 with NaF, versus 52 % by day 14 without NaF.

On the basis of these experimental data, sampling of VH from each eyeball, without pooling, would seem to be a good compromise. One sample, dedicated to toxicology analysis, should be performed with a stabilizer (1.5 % NaF or KF) to prevent ethanol neoformation and degradation of xenobiotics such as benzodiazepines, 6-MAM or cocaine. The other sample, without stabilizer, serves for biochemistry analysis. Both samples should be stored at −20 °C.

### Sample preparation

The composition of VH makes it relatively “clean” in comparison to other autopsy matrices, and analysis does not require complex preparation. Some assays do not include an extraction step. Davis et al. [97] described the analysis of four antibiotics (fluoroquinolones) by direct injection using high-performance liquid chromatography/ultraviolet (HPLC/UV) and HPLC/fluorescence detection. Logan and Stafford [90] developed an HPLC neuroleptic assay based on injection after dilution and filtration using a preconcentration column. A similar process was also proposed for cocaine and benzoylecgonine [98].

The extraction techniques generally used for blood and other fluids provide cleaner extracts of VH than other matrices. Solid-phase extraction (SPE) is widely used, notably for assaying benzodiazepines [99], drugs of abuse (e.g., opiates, methadone, cocaine,) [100–104] and paracetamol [103]. Bévalot et al. [105] described a generic technique, validated on six compounds and assessed for implementation in large-scale screening. Liquid/liquid extraction (LLE) was used to assay colchicine [106], clotiapine [107], benzodiazepines [108], memantine after 9-fluorenylmethyl-chloroformate-chloride derivation [109], beta-blockers using an Extrelut^®^ column [110], narcotics [111], arylcyclohexylamines (methoxetamine, 3-methoxyeticyclidine and 3-methoxyphencyclidine) [112] and LSD [113]. For narcotics analysis, microwave-assisted LLE provided better recovery and precision than SPE [114]. Other, less widespread techniques have also been tested. Disposable pipette extraction showed recovery in a range of 72–91 % for opiates, with a volume of 100 μL required for the VH sample [115]. Supercritical-phase extraction, which limits the need for an organic solvent, has been used with success in opiate assay [115, 116].

Several authors have recommended liquefying VH samples before analysis by immunoassay analyzers in order to avoid the phenomenon of clogging due to viscosity. Liquefaction techniques include enzymatic hydrolysis by hyaluronidase, heating, microfiltration, dilution and centrifugation [86].

### Analytical techniques

Instrumental techniques for analysis have greatly improved in sensitivity and selectivity. Unlike more complex postmortem matrices (e.g., blood, tissue), where enhanced selectivity serves to palliate interference, VH, which can be considered a “clean” matrix, benefits fully from the gain in sensitivity. Analysis of cocaine and cocaine derivatives is a good example illustrating the whole range of analytical techniques that have been implemented for VH analysis: gas chromatography/flame ionization detector (GC/FID) [104], high-performance liquid chromatography/diode array detector (HPLC/DAD) [100, 114], GC/mass spectrometry (MS) [101, 117, 118], GC/tandem mass spectrometry (MS–MS) [95], HPLC/MS–MS [119], capillary electrophoresis/DAD [111] and immunoanalysis (cloned enzyme donor immunoassay [120], enzyme multiplied immunoassay technique [59]). Few screening techniques seem to have been specifically dedicated to VH analysis. Pelander et al. [121] described an assay based on HPLC coupled with time-of-flight mass spectrometry that allowed the detection of 70 compounds, and more recently, a method for the simultaneous screening and quantification of 24 analytes of forensic interest was described by Arora et al. [122].

## Interpretation of vitreous humor analysis results

In 1969, Felby and Olsen [51] published one of the first reports of postmortem medical drug analysis in VH. In this study, VH concentrations of barbiturates in a given individual were identical to blood ultrafiltrate levels but different from those in total blood, showing that barbiturates penetrate VH by passive diffusion. The authors also suggested an effect of plasma protein binding on VH penetration in certain barbiturates (phenobarbital, aprobarbital and barbital). No significant differences in concentration were found between left and right VH drug levels. The authors suggested that an ultrafiltrate/VH concentration ratio >1 indicated death earlier than the equilibrium phase, and thus shortly after intake. They considered analysis technically easier in VH than in blood, especially in the case of putrefaction. Thus, from its earliest applications in postmortem toxicology, various advantages of VH analysis have been highlighted, and most have since been studied in numerous medical and narcotic drug families.

### Case reports

Table 2 [60, 101, 103, 106, 107, 113, 123–256] presents cause of death and blood and VH concentrations from more than 100 case reports. It is intended as a practical tool for toxicologists in analyzing and interpreting results for specific compounds, which were arranged in the alphabetical order.

**Table 2:** Case reports providing vitreous humor concentrations: substance name, number of cases reported (*n*), cause of death, blood concentrations (peripheral and/or cardiac), vitreous humor (VH) concentration (ND: not detected, NQ: detected but not quantified) and reference

Substance	*N*	Cause of death	Blood concentration		VH concentration Mean (range)	Reference(s)
			Peripheral blood	Cardiac blood		
25C-NBOMe	1	Fatal poisoning involving 25C-NBOMe	0.60 µg/kg (antemortem: 0.81 µg/kg)	–	0.33 µg/kg	[123]
25I-NBOMe	1	Fatal intoxication involving 25I-NBOMe	405 pg/mL	410 pg/mL	99 pg/mL	[124]
4-MTA	1	Overdose fatality involving 4-MTA and MDMA	5.49 mg/L	7.60 mg/L	1.31 mg/L	[125]
5-(2-Aminopropyl) indole (5-IT)	2	Fatal intoxication due to 5-IT	Preserved: 1.2 mg/L Unpreserved: 0.8 mg/L	1.2 mg/L	0.8 mg/L	[126]
		Multiple drug intoxication	Preserved: 1.0 mg/L Unpreserved: 0.9 mg/L	2.6 mg/L	1.4 mg/L	
6-MAM	2	Multiple drug intoxication	Blood: 22.0 ng/mL (0.93–21.1)		66.0 ng/mL (26.8–131.92)	[127]
Acebutolol	1	Fatal intoxication involving acebutolol	Blood: 34.7 µg/mL		17.9 µg/mL	[128]
Acetaminophen	2	Possible cardiac mechanism of death associated with high level of acetaminophen	1280 mg/L	–	878 mg/L	[129]
			–	1220 mg/L	779 mg/L	
Acetaminophen	1	Multiple drug intoxication	Left: 60 mg/L Right: 60 mg/L	Thoracic: 30 mg/L	57 mg/L	[130]
Acetone	1	Multiple drug intoxication	103 mg/100 mL	77 mg/100 mL	120 mg/100 mL	[131]
Aconitine	1	Suicidal Aconitum poisoning	17.9 µg/L	87.9 µg/L	8.4 µg/L	[132]
Alprazolam	1	Suicide by acute alprazolam overdose	2.3 mg/L	2.1 mg/L	0.58 mg/L	[133]
Amitriptyline	1	Fatal self-poisoning involving amitriptyline	Blood: 0.82 mg/L		6.05 mg/L	[134]
Amitriptyline (nortriptyline)	1	Fatal intoxication involving amitriptyline and nortriptyline	Plasma: 0.25 µg/mL (nortriptyline: 0.58 µg/mL)		0.05 µg/mL (nortriptyline: 0.06 µg/mL)	[135]
Amitriptyline (nortriptyline)	1	Fatal intoxication involving amitriptyline and nortriptyline	1.8 µg/mL (nortriptyline: 0.6 µg/mL)	Superior vena cava: 2.8 µg/mL (nortriptyline: 1.2 µg/mL)	0.8 µg/mL (nortriptyline: ND)	[136]
Amitriptyline (nortriptyline)	1	Multiple drug intoxication	2.5 mg/L (nortriptyline: 0.7 µg/mL)	Superior vena cava: 7.1 mg/L (nortriptyline: 0.9 µg/mL)	0.67 mg/L	[137]
Amobarbital	2	Deaths induced by or related to drug	Blood: 6 mg/L		8 mg/L	[138]
			Blood: 28 mg/L		26 mg/L	
Amoxapine	1	Suicide by amoxapine intoxication	Blood: 11.50 mg/L		0.20 mg/L	[139]
Amphetamine	1	Suicide by methamphetamine overdose	0.74 mg/L	–	0.27 mg/L	[140]
Amphetamine	1	Fatal intoxication after massive methamphetamine ingestion	0.43 mg/L	0.70 mg/L	0.64 mg/L	[141]
Amphetamine	1	Accidental death due to methamphetamine	1.3 mg/L	–	0.5 mg/L	[142]
Arsenic	1	Fatal intoxication due to arsenic ingestion	Blood: 1.3 mg/L		0.050 mg/L	[143]
Atomoxetine	2	Arrhythmogenic right ventricular dysplasia	0.33 mg/L	0.65 mg/L	0.1 mg/L	[144]
		Suicide by venlafaxine and atomoxetine overdose	5.4 mg/L	8.3 mg/L	0.96 mg/L	
Benzphetamine	1	Suicide by benzphetamine intoxication	Blood: 13.9 µg/mL		21.0 µg/mL	[145]
Brodifacoum	1	Fatal intoxication involving brodifacoum	3920 ng/mL	–	ND	[146]
Bupivacaine	1	Fatal intoxication involving bupivacaine	3.8 mg/L	2.8 mg/L	1.3 mg/L	[147]
Butriptyline	1	Suicide by butriptyline intoxication	Blood: 14.9 mg/L		0.52 mg/L	[148]
Caffeine	3	Accidental fatal overdose	Blood: 184.1 mg/L		99.8 mg/L	[149]
		Suicide by overdose	Blood: 343.9 mg/L		95.9 mg/L	
		Suicide by overdose	Blood: 251.0 mg/L		147 mg/L	
Caffeine	1	Multiple drug intoxication	Blood: 3000 ng/mL		1550 ng/mL	[127]
Carbon tetrachloride	1	Fatal intoxication after carbon tetrachloride ingestion	143 mg/L	57.5 mg/L	170 mg/L	[150]
Chloralose	1	Fatal intoxication involving chloralose	65.1 mg/L		24.7 mg/L	[151]
Chlorpheniramine	1	Multiple drug intoxication	Blood: 0.2 mg/L		0.1 mg/L	[152]
Chlorpyrifos-methyl	1	Fatal intoxication involving chlorpyrifos-methyl	0.615 mg/L	Cardiac chambers: Left = 1.01 mg/L Right = 1.71 mg/L	0.009 mg/L	[153]
Citalopram	9	Fatal intoxication involving citalopram	0.8 mg/L	–	0.3 mg/L	[154]
		Multiple drug intoxication	0.4 mg/L (0.2–0.7)	–	0.23 mg/L (0.1–0.4)	
		Other	0.28 mg/L (0.1–0.4)	–	0.14 mg/L (0.1–0.2)	
Citalopram	1	Multiple drug intoxication	Blood: 758 ng/mL		1130 ng/mL	[127]
Citalopram	1	Fatal intoxication involving cyproheptadine and citalopram	2.3 mg/L	–	0.8 mg/L	[155]
Clomipramine	1	Fatal intoxication involving clomipramine	Blood: 1729 ng/mL		1000 ng/mL	[156]
Clotiapine	3	Acute mixed intoxication	110 µg/L	75 µg/L	16 µg/L	[107]
		Unknown	310 µg/L	–	19 µg/L	
		Acute mixed intoxication	340 µg/L	200 µg/L	30 µg/L	
Clozapine	1	Suicide by acute clozapine overdose	8.8 mg/L	12.0 mg/L	1.3 mg/L	[157]
Cocaine	2	Fatal intoxication involving cocaine	Blood: 1.8 mg/L		2.4 mg/L	[158]
			Blood: 13.0 mg/L		14.0 mg/L	
Cocaine	1	Overdose fatality involving cocaine	Blood: 330 mg/L		13 mg/L	[159]
Cocaine	1	Cocaine poisoning in a body packer	4 µg/mL		7.1 µg/mL	[160]
Cocaine	1	Asphyxiation by hanging	3210 ng/mL	Left: 1640 ng/mL Right: 1110 ng/mL	230 ng/mL	[101]
Cocaine	1	Swallowing of a bag of cocaine	Blood: 211 mg/L		0.8 mg/L	[161]
Cocaine	3	Fatal intoxication due to cocaine	Blood: 0.37 mg/dL		0.21 mg/dL	[162]
			Blood: 0.75 mg/dL		0.38 mg/dL	
			Blood: 0.11 mg/dL		0.14 mg/dL	
Cocaine (BZE, EME)	1	Cocaine overdose	5.0 mg/L (BZE = 10.4 mg/L; EME = 4.1 mg/L)	9.0 mg/L (BZE = 20.1 mg/L; EME = 14.4 mg/L)	5.3 mg/L (BZE = 5.6 mg/L; EME = 2.6 mg/L)	[163]
Cocaine (BZE)	4	Not specified	Blood: <5 ng/mL (BZE: <5 ng/mL)		<5 ng/mL (BZE: 30 ng/mL)	[60]
		Not specified	Blood: <5 ng/mL (BZE: 216 ng/mL)		Traces (BZE: 311 ng/mL)	
		Not specified	Blood: 400 ng/mL (BZE: 800 ng/mL)		250 ng/mL (BZE: 420 ng/mL)	
		Not specified	Blood: 5000 ng/mL (BZE: 90 ng/mL)		2300 ng/mL (BZE: 120 ng/mL)	
Codeine	3	Multiple drug intoxication	Blood: 30.92 ng/mL (18.6–49.18)		26.27 ng/mL (15.3–32.5)	[127]
Codeine	1	Unknown	Total: 1280 ng/mL Fee: 117 ng/mL	Total: 1260 ng/mL Free: 212 ng/mL	Total: 799 ng/mL Free: 342 ng/mL	[103]
Codeine	11	Not specified	Blood: <5 ng/mL		<5 ng/mL	[60]
		Not specified	Blood: <5 ng/mL		61 ng/mL (36–86)	
		Not specified	Blood: 30 ng/mL (20–40)		< 5 ng/mL	
		Not specified	Blood: 333 ng/mL (100–500)		77 ng/mL (20–150)	
Codeine (6-glucuronide; norcodeine)	2	Not specified	221 ng/mL (6-glucuronide = 3530 ng/mL; norcodeine = 17 ng/mL)	223 ng/mL (6-glucuronide = 2170 ng/mL; norcodeine = 19 ng/mL)	279 ng/mL (6-glucuronide = 185 ng/mL; norcodeine = 9 ng/mL)	[164]
		Not specified	8770 ng/mL (6-glucuronide = 17,100 ng/mL; norcodeine = 500 ng/mL)	1580 ng/mL (6-glucuronide = 2179 ng/mL; norcodeine = 73 ng/mL)	1180 ng/mL (6-glucuronide = 1230 ng/mL; norcodeine = 25 ng/mL)	
Colchicine	2	Suicidal colchicine poisoning	17.4 ng/mL	5.2 ng/mL	3 ng/mL	[165]
			21.9 ng/mL	22.8 ng/mL	0.5 ng/mL	
Colchicine	1	Fatal accidental intoxication by colchicine	–	50 µg/L	10 µg/L	[106]
Colchicine	1	Fatal overdose involving colchicine	29 ng/mL	–	<5 ng/mL	[166]
Cyanide	1	Fatal intoxication involving potassium cyanide	Blood: 21.5 mg/L		1.3 mg/L	[167]
Cyproheptadine	1	Fatal intoxication involving cyproheptadine and citalopram	0.49 mg/L	–	<0.04 mg/L	[155]
Dextromethorphan	1	Multiple drug intoxication	Blood: 41.5 ng/mL		12 ng/mL	[127]
Dichlorvos	1	Fatal intoxication involving dichlorvos	ND	Cardiac chambers: Left = ND; Right = ND	0.067 mg/L	[153]
Digoxin	4	Unknown	Blood: 0.01 µg/mL		0.001 µg/mL	[168]
			Blood: 0.012 µg/mL		0.009 µg/mL	
			Blood: 0.039 µg/mL		0.003 µg/mL	
			Blood: 0.098 µg/mL		0.048 µg/mL	
Diltiazem	1	Suicide by diltiazem intoxication	Blood: 6.7 mg/L		5.5 mg/L	[169]
Diphenhydramine	1	Homicide by acute diphenhydramine intoxication	Blood: 1.6 mg/L		0.7 mg/L	[170]
Diphenhydramine	1	Multiple drug intoxication	Blood: 8.8 mg/L		1 mg/L	[152]
Dizocilpine (MK-801)	1	Multiple drug intoxication	Blood: 0.15 mg/L		<0.1 mg/L	[171]
Duloxetine	*5*	Diabetic ketoacidosis	ND	+<0.05 mg/L	ND	[172]
		Morphine intoxication	–	0.22 mg/L	0.06 mg/L	
		Methadone intoxication	0.20 mg/L	0.23 mg/L	0.09 mg/L	
		Multiple drug intoxication	0.19 mg/L	0.30 mg/L	0.11 mg/L	
		Poly-med overuse	0.26 mg/L	0.59 mg/L	0.23 mg/L	
Embutramide	1	Suicide by Tanax (embutramide, mebezonium iodide and tetracaine) injection	5.06 mg/L	–	2.74 mg/L	[173]
Ethyl chloride	1	Fatal intoxication involving multiple drug	Blood: 423 mg/L		12 mg/L	[174]
Ethyl chloride	1	Overdose or adverse reaction to ethyl chloride	Blood: 65 mg/dL		41.7 mg/dL	[175]
Ethyltryptamine	1	Fatal intoxication involving ethyltryptamine	–	5.6 mg/L	2.4 mg/L	[176]
Etomidate	3	Suicide by intoxication by etomidate	0.40 mg/L	–	0.30 mg/L	[177]
		Medical intervention/crush injuries	0.05 mg/L	–	<0.026 mg/L	
		Medical intervention/injury at chest and abdomen	<0.026 mg/L	–	0.04 mg/L	
Fentanyl	4	Bronchopneumonia, pulmonary and aortic thrombosis	–	1.8 µg/L	+< 2.0 µg/L	[178]
		Fatal intoxication involving fentanyl	4.5 µg/L	6.4 µg/L	8.0 µg/L	
		Pneumonia	6.8 µg/L	4.8 µg/L	10 µg/L	
		Pleural mesothelioma (intake of analgesia)	18 µg/L	16 µg/L	20 µg/L	
Fentanyl	1	Fatal intoxication involving fentanyl	Left: 20.9 µg/L Right: 21.3 µg/L	Left: 33.9 µg/L Right: 37.6 µg/L	19.5 µg/L	[179]
Fentanyl	1	Suicidal intoxication by fentanyl	94.9 ng/g	Left: 45.9 ng/g Right: 74.8 ng/g	133 ng/g	[180]
Flecainide	1	Fatal intoxication involving flecainide	Blood: 13 mg/L		7.4 mg/L	[181]
Fluoride	1	Suicide due to fluoride poisoning	19.4 mg/L	–	2.5 mg/L	[182]
Fluoxetine	3	Civil aviation accident	Blood: 0.057 µg/mL		0.005 µg/mL	[183]
			Blood: 0.338 µg/mL		0.024 µg/mL	
			Blood: 0.280 µg/mL		0.038 µg/mL	
Flurazepam	1	Suicide by acute flurazepam overdose	5.5 mg/L	–	1.3 mg/L	[184]
Fluvoxamine	3	Accidental asphyxia by choking	0.49 mg/L	–	0.16 mg/L	[185]
		Suicide by shotgun wound to chest	0.48 mg/L	1.5 mg/L	0.28 mg/L	
		Suicide by fluvoxamine intoxication	5.9 mg/L	–	1.9 mg/L	
GHB	1	Fatal intoxication GHB/heroin	11.5 µg/mL		84.3 µg/mL	[186]
GHB	1	Fatal overdose involving GHB	2937 mg/L	3385 mg/L	2856 mg/L	[187]
GHB	1	Fatal GHB intoxication	461 mg/L	276 mg/L	48 mg/L	[188]
Guaifenesin	1	Multiple drug intoxication	Blood: 27.4 mg/L		7 mg/L	[152]
Hydromorphone (3-glucuronide)	1	Acute aspiration-related bronchopneumonia, secondary to hydromorphone ingestion	57 ng/mL (hydromorphone-3-glucuronide: 459 ng/mL)	–	31 ng/mL (hydromorphone-3-glucuronide: 40 ng/mL)	[189]
Imipramine	1	Multiple drug intoxication	Left: 2.3 mg/L Right: 2.5 mg/L	Thoracic: 5.2 mg/L	1.4 mg/L	[130]
Insulin	1	Fatal intoxication involving insulin (death 4 days after insulin administration)	–	–	Approximately 1.0 ng/mL (24.8 µIU/mL)	[190]
Insulin	1	Suicidal insulin administration	Serum: 583 mU/L	–	11.5 mU/L	[191]
Insulin	1	Suicide by insulin self injection	ND	ND	103 µU/mL	[192]
Insulin	1	Possible suicidal poisoning involving insulin	–	–	24.4 µIU/mL	[193]
Insulin	1	Self-injected insulin overdose	–	–	31µU/mL	[194]
Lamotrigine	5	Epilepsy	Blood: 12.9 mg/L (0.9–38)		4.62 mg/L (0.3–14)	[195]
Lithium	1	Mixed-drug intoxication involving tranylcypromine and lithium	0.57 µmol/L	–	0.79 µmol/L	[196]
Loxapine	1	Suicide by acute loxapine overdose	–	9.5 mg/L	1.5 mg/L	[197]
LSD	1	Not specified	Blood: 3.2 ng/mL		2.9 ng/mL	[113]
mCPP	1	Fatal intoxication involving mCPP	Embalmed		4.7 ng/mL	[198]
MDMA	1	Acute cardiopulmonary failure	3.1 µg/mL	5.7 µg/mL	3.4 µg/mL	[199]
MDMA	1	Fatal intoxication involving MDMA	Blood: 2.9 mg/L		1.9 mg/mL	[200]
MDMA	1	Overdose fatality involving 4-MTA and MDMA	10.5 µg/L	16.5 µg/L	67.6 µg/L	[125]
MDMA	1	Fatal hyperthermia	–	0.42 µg/mL	0.361 µg/mL	[201]
Mephedrone	1	Fatal intoxication involving mephedrone	Blood: 5.5 µg/mL		7.1 µg/mL	[202]
Mescaline	1	Multiple gunshots wounds	Blood: 2.95 mg/L		2.36 mg/L	[203]
Methadone	1	Fatal intoxication involving methadone	Subclavian: 0.67 mg/L	–	0.24 mg/L	[204]
Methadone (EDDP)	3	Not specified	Blood: <5 ng/mL (EDDP < 5 ng/mL)		21 ng/mL (EDDP: 55 ng/mL)	[60]
		Not specified	Blood: <5 ng/mL (EDDP <5 ng/mL)		28.6 ng/mL (EDDP: 53.9 ng/mL)	
		Not specified	Blood: <5 ng/mL (EDDP <5 ng/mL)		36 ng/mL (EDDP: 74 ng/mL)	
Methadone	2	Death induced by or related to drug	Blood: 1.0 mg/L		82 ng/L	[138]
		Death induced by or related to drug	Blood: 1.4 mg/L		50 ng/L	
Methamphetamine	1	Suicide by methamphetamine overdose	30 mg/L	–	7.1 mg/L	[140]
Methamphetamine	1	Fatal intoxication after a massive methamphetamine ingestion	53.7 mg/L	65.7 mg/L	45.8 mg/L	[141]
Methamphetamine	1	Accidental death due to methamphetamine	42.6 mg/L	–	20.1 mg/L	[142]
Methanol	1	Fatal intoxication involving methanol	Blood: 142 mg/dL		173 mg/dL	[205]
Methanol	1	Homicidal poisoning by methanol	0.23 % (w/v)	0.21 % (w/v)	0.28 % (w/v)	[206]
Methanol	1	Suicide by methanol ingestion	Blood: 2.84 g/L		3.96 g/L	[207]
Methanol	3	Fatal intoxication involving methanol	5 mg/L	5 mg/L	8 mg/L	[208]
			228 mg/L	254 mg/L	201 mg/L	
			2070 mg/L	2130 mg/L	2120 mg/L	
Methanol	1	Impact trauma and methanol poisoning	31.2 mg/dL	–	ND	[209]
Methanol	44	Fatal methanol poisoning	Blood: 150 ± 143 mg/dL		155 ± 144 mg/dL	[210]
Methanol	1	Fatal intoxication due to methanol ingestion	Blood: 0.1 g/L (100 mg/L)—1 h before death and 61 h after hospitalization Formic acid: 5.1 mg/L at the same time		120 mg/L Formic acid: 21.3 mg/L	[211]
Methomyl	1	Respiratory paralysis	3 ng/mL	Left: 8 ng/mL Right: 6 ng/mL	2680 ng/mL	[212]
Methylone	1	Fatal intoxication involving methylone	3.4 mg/L	3.4 mg/L	4.3 mg/L	[213]
Methylphenidate	1	Fatal intoxication involving methylphenidate	1.1 mg/L	0.98 mg/L	0.80 mg/L	[214]
Metoprolol	1	Suicide by acute metoprolol overdose	Blood: 19.8 mg/L		15.1 mg/L	[215]
Metoprolol	1	Suicide by acute metoprolol overdose	Blood: 4.7 mg/L		3.3 mg/L	[216]
Mexiletine	1	Suicide by acute mexiletine overdose	14 mg/L	38 mg/L	17 mg/L	[217]
Mexiletine	1	Fatal overdose of mexiletine	10.0 µg/mL	44.8 µg/mL	8.6 µg/mL	[218]
Mirtazapine	3	Therapeutic use of mirtazapine	–	0.21 mg/L	0.06 mg/L	[219]
			0.22 mg/L	0.31 mg/L	0.09 mg/L	
			0.24 mg/L	0.32 mg/L	0.10 mg/L	
Mirtazapine	6	Suicide by multiple drug intoxication	2.1 mg/L	2.3 mg/L	1.0 mg/L	[220]
		Suicide by mirtazapine and desipramine intoxication	3.4 mg/L	2.0 mg/L	1.2 mg/L	
		Accidental multiple drug intoxication	0.45 mg/L	0.38 mg/L	0.14 mg/L	
		Hypertensive	0.44 mg/L	0.36 mg/L	0.30 mg/L	
		Suicide by asphyxia	0.08 mg/L	0.08 mg/L	0.04 mg/L	
		Other	0.03 mg/L	0.04 mg/L	0.01 mg/L	
Mitragynine	1	Multiple drug intoxication	0.23 mg/L	0.19 mg/L	<0.05 mg/L	[221]
Moclobemide	1	Multiple drug intoxication	21 mg/L	–	11 mg/L	[222]
Morphine	1	Fatal intoxication GHB/heroin	0.77 µg/mL		0.3 µg/mL	[186]
Morphine	1	Fatal intoxication involving heroin and ethanol	Blood: 0.68 µg/mL		0.062 µg/mL	[223]
Morphine	1	Fatal accidental intoxication involving morphine	Blood: unconjugated: 0.460 mg/L Total: 0.624 mg/L		Unconjugated: 0.034 mg/L Total: 0.08 mg/L	[224]
Morphine	3	Multiple drug intoxication	Blood: 22.8 ng/mL (110–307.82)		232 ng/mL (151.1–328)	[127]
Morphine	1	Unknown	Total: 270 ng/mL Free: ND	Total: 397 ng/mL Free: ND	Total: 162 ng/mL Free: ND	[103]
Morphine	10	Not specified	Blood: <5 ng/mL		52 ng/mL	[60]
		Not specified	Blood: <5 ng/mL		25 ng/mL	
		Not specified	Blood: 130 ng/mL (20–200)		77 ng/mL (20–169)	
Morphine	2	Fatal overdose involving heroin	Blood: 0.021 mg/L		0.353 mg/L	[225]
		Fatal overdose involving heroin	Blood: 0.173 mg/L		0.030 mg/L	
Morphine	1	Fatal ingestion of 75 packets of heroin	Aortic blood: 0.68 mg/L		0.17 mg/L	[226]
Morphine	13	Death induced by or related to drug	Blood: 0.078 mg/L (0.03–0.14)		1.20 mg/L(0.05–4.20)	[138]
		Death induced by or related to drug	Blood: ND		0.03 mg/L	
		Fatal intoxication involving opiates	Blood: 1.70 mg/L		ND	
Morphine (3-glucuronide; 6-glucuronide)	2	Not specified	3 ng/mL (3-glucuronide = 125 ng/mL; 6-glucuronide <23 ng/mL)	3 ng/mL (3-glucuronide = 111 ng/mL; 6-glucuronide <23 ng/mL)	2 ng/mL (3-glucuronide <23 ng/mL; 6-glucuronide 0 ng/mL)	[164]
		Not specified	521 ng/mL (3-glucuronide = 1860 ng/mL; 6-glucuronide = 606 ng/mL)	114 ng/mL (3-glucuronide = 328 ng/mL; 6-glucuronide = 75 ng/mL)	34 ng/mL (3-glucuronide = 161 ng/mL; 6-glucuronide = 57 ng/mL)	
Nefopam	1	Fatal overdose due to nefopam Atherosclerotic coronary artery disease	Preserved: 14.7 mg/L Unpreserved: 13.6 mg/L	Unpreserved: 21.2 mg/L	Preserved: 4.5 mg/L	[227]
Nicotine	1	Respiratory paralysis	222 ng/mL	Left: 733 ng/mL Right: 666 ng/mL	234 ng/mL	[212]
Nicotine	1	Asphyxiation	0.46 µg/mL	1.4 µg/mL	0.27 µg/mL	[228]
Olanzapine	1	Hypertensive cardiovascular disease	–	550 ng/mL	ND	[229]
Oxcarbazepine	1	Possible intoxication involving oxcarbazepine	2.9 mg/kg	–	ND	[230]
Oxycodone	7	Hypertrophic cardiomyopathy, probable effect of oxycodone toxicity	0.19 mg/L	0.29 mg/L	0.4 mg/L	[231]
		Hypertrophic cardiomyopathy	0.12 mg/L	0.18 mg/L	0.18 mg/L	
		Effects of acute and chronic narcotic addiction	0.35 mg/L	0.12 mg/L	0.24 mg/L	
		Pneumonia, oxycodone toxicity	1.5 mg/L	1.2 mg/L	0.25 mg/L	
		Cardiomyopathy	–	0.27 mg/L	0.32 mg/L	
		Multiple drug intoxication	–	0.75 mg/L	0.51 mg/L	
		Acute ingestion of multiple oral medications	0.59 mg/L	0.82 mg/L	0.82 mg/L	
Oxycodone	2	Suicide by oxycodone intoxication	Blood: 3.6 mg/L		2.1 mg/L	[232]
Oxycodone		Fatal intoxication involving oxycodone	Blood: 0.76 mg/L		0.63 mg/L	
Pentobarbital	1	Suicide by acute pentobarbital overdose	13.5 µg/mL	–	12.6 µg/mL	[233]
Pentobarbital	5	Death induced by or related to drug	Blood: 19.4 mg/L (3–41)		15 mg/L	[138]
Phenazepam (3-OH-phenazepam)	24	Multiple drug intoxication (*n* = 21)	Preserved: 0.019 mg/L (0.011–0.360) (3-OH-phenazepam: 0.070 mg/mL [ND–0.246])	Unpreserved: 0.108 mg/L (0.014–0.310) (3-OH-phenazepam: 0.063 mg/mL [ND–0.161])	Preserved: 0.018 mg/L (<0.007–0.054) (3-OH-phenazepam: 0.05 mg/mL [ND–0.05])	[234]
		Hanging (*n* = 2)	Preserved: 0.069 mg/L (0.007–0.131) (3-OH-phenazepam: <0.016 mg/mL)	Unpreserved: 0.138 mg/L (3-OH-phenazepam: <0.016 mg/mL)	Preserved: 0.013 mg/L (ND–0.013) (3-OH-phenazepam: <0.016 mg/mL)	
		Pulmonary thromboembolism (*n* = 1)	Preserved: 0.103 mg/L (3-OH-phenazepam: 0.022 mg/mL)	Unpreserved: 0.074 mg/L (3-OH-phenazepam: 0.020 mg/mL)	Preserved: 0.008 mg/L (3-OH-phenazepam: <0.016 mg/mL)	
Phenobarbital	6	Death induced by or related to drug	Blood: 15.8 mg/L (4–25)		10.2 mg/L	[138]
Propylhexedrine	3	Fatal overdose of propylhexedrine	Blood: 0.16 µg/mL		2.2 µg/mL	[235]
			Blood: 0.3 µg/mL		0.5 µg/mL	
			Blood: 9.4 µg/mL		1.1 µg/mL	
Quetiapine	6	Acute combined ethanol and quetiapine poisoning	6.0 mg/L	–	1.0 mg/L	[236]
		Suicide by quetiapine overdose	1.0 mg/L	–	1.0 mg/L	
		Suicide by mixed-drug overdose	7.0 mg/L	–	1.4 mg/L	
		Fatal mixed-drug overdose	0.40 mg/L	–	0.20 mg/L	
		Acute myocardial ischemia due to coronary artery atherosclerosis	1.0 mg/L	–	<0.40 mg/L	
		Suicide by quetiapine overdose	10.2 mg/L	–	3.2 mg/L	
Quetiapine	2	Suicide by quetiapine intoxication	–	7.20 mg/L	0.93 mg/L	[237]
			–	16 mg/L	1.8 mg/L	
Quetiapine	5	Cocaine intoxication	Blood: detected		<0.05 mg/L	[238]
		Multiple drug intoxication	Blood: 2.7 mg/L		0.11 mg/L	
		Multiple drug intoxication	Blood: 1.3 mg/L		0.08 mg/L	
		Other	Blood: 0.15 mg/L		0.06 mg/L	
		Other	Blood: 0.37 mg/L		0.15 mg/L	
Ricinine	1	Suicide by injection of castor bean extract	Blood: 2.3 ng/mL		NQ	[239]
Ropinirole	1	Fatal intoxication involving ropinirole	64 ng/mL	–	11 ng/mL	[240]
Salicylate	1	Multiple drug intoxication	81 mg/L	Superior vena cava: 148 mg/L	42 mg/L	[137]
Secobarbital	7	Death induced by or related to drug	Blood: 11.9 mg/L (1–28)		5.4 mg/L	[138]
Sertraline	4	Civil aviation accident	Blood: 0.302 µg/mL		0.004 µg/mL	[241]
			Blood: 0.064 µg/mL		0.001 µg/mL	
			Blood: 0.240 µg/mL		0.007 µg/mL	
			Blood: 0.143 µg/mL		0.001 µg/mL	
Sertraline	1	Multiple drug intoxication	0.9 mg/L	–	0.5 mg/L	[222]
Strychnine	1	Suicide by rodenticide poisoning	0.96 mg/L	0.31 mg/L	0.36 mg/L	[242]
Sufentanil	1	Suicide by intoxication involving sufentanil and midazolam	Blood: 1.1 ng/mL		1.2 ng/mL	[243]
THC-COOH	50	Automobile accident involving marijuana intake	Blood: 0.081 µg/mL (0.016–0.330)		Detected *n* = 39 Mean: <0.010 µg/mL	[244]
Topiramate	1	Seizure disorder with upper respiratory infection	Blood: 8.9 mg/L		12.4 mg/L	[245]
Tranylcypromine	1	Mixed-drug intoxication involving tranylcypromine and lithium	0.19 µg/mL	–	0.22 µg/mL	[196]
Triazolam	1	Postural asphyxia caused by triazolam poisoning	Right femoral vein: 62 ng/mL	Left chamber: 90 ng/mL Right chamber: 153 ng/mL	Right VH: 19 ng/mL	[246]
Tripelennamine	1	Fatal intoxication involving tripelennamine	Blood: 1.0 mg/100 mL (10 µg/mL)		43 µg/mL	[247]
Valproic acid	1	Fatal intoxication involving valproic acid	Blood: 1050 mg/mL		516 mg/mL	[248]
Varenicline	1	Fatal overdose of varenicline	Subclavian: 262 ng/mL Femoral: 257 ng/mL	–	165 ng/mL	[249]
Venlafaxine	1	Suicide by mixed-drug intoxication	6.2 mg/L	–	5.3 mg/L	[250]
Venlafaxine	3	Suicide by venlafaxine overdose (combined drug toxicity)	7.2 mg/L	–	4.8 mg/L	[251]
			31 mg/L	–	31 mg/L	
			36 mg/L	–	10 mg/L	
Venlafaxine	9	Other	1.7 mg/L (0.1–6.6)	–	1.08 mg/L (<0.05–3.6)	[251]
Venlafaxine	2	Suicide by multiple drug intoxication	17 mg/L	30 mg/L	11 mg/L	[252]
		Suicide by venlafaxine intoxication	65 mg/L	85 mg/L	23 mg/L	
Venlafaxine	2	Fatal overdose due to venlafaxine	Blood: 53 mg/L		22 mg/L	[253]
		Fatal overdose due to venlafaxine	Blood: 78 mg/L		58 mg/L	[253]
Verapamil	1	Suicide by mixed-drug intoxication	3.5 mg/L	–	1.0 mg/L	[250]
Zipeprol	1	Fatal overdose involving zipeprol	–	6.69 mg/L	6.08 mg/L	[254]
Zolpidem	2	Suicide by acute zolpidem overdose	Blood (iliac) = 1.6 mg/L		0.52 mg/L	[255]
			Blood (subclavian = 4.5 mg/L); (iliac = 7.7 mg/L)		1.6 mg/L	
Zopiclone	1	Suicide by acute zopiclone overdose	254 ng/mL	408 ng/mL	94 ng/mL	[256]

The expressions (unit, number of decimals) of concentrations reported in the table are identical to those published in the original articles

*25*-*NBDMe* 2-(4-chloro-2,5-dimethoxyphenyl)-*N*-[(2-methoxyphenyl)methyl]ethanamine; *25I*-*NBOMe* 2-(4-iodo-2,5-dimethoxyphenyl)-*N*-[(2-methoxyphenyl)methyl]ethanamine; *4*-*MTA* 4-methylthioamphetamine; *6*-*MAM* 6-monoacetylmorphine; *BZE* benzoylecgonine; *EME* ecgonine methyl ester; *GHB* Gamma-hydroxybutyric acid; *LSD* Lysergic acid diethylamide; *mCPP* meta-chlorophenylpiperazine; *MDMA* 3,4-methylenedioxymethamphetamine; *EDDP* 2-ethylidene-1,5-dimethyl-3,3-diphenylpyrrolidine; *THC*-*COOH* 11-nor-9-carboxy-delta-9-tetrahydrocannabinol; *ND* not detectable; *NQ* not quantified

### Qualitative interpretation

Toxicologic analysis of VH is of undisputed qualitative interest, as seen from the large number of xenobiotics detected (Table 2). Its qualitative importance compared to blood and other matrices has been assessed for various groups of compounds.

#### Opiates and opioids

Interpretation of recent heroin intake via its tracer 6-MAM has been a particular focus of study. In a series of 29 deaths from opiates, Pragst et al. [257] reported that in two cases, 6-MAM was detected in VH but not in urine, despite generally higher 6-MAM concentrations in urine than in VH. Blood concentrations of 6-MAM were not reported in this study. Wyman and Bultman [258], Rees et al. [259], Antonides et al. [118] and Scott et al. [116] showed that in heroin-related deaths, if only blood were analyzed, 6-MAM would go undetected in 36 % (*n* = 25), 59 % (*n* = 70), 50 % (*n* = 12) and 25 % (*n* = 20) of cases, respectively. Two hypotheses have been put forward to explain why 6-MAM should be detected more often in VH than in blood: good membrane crossing due to lipophilicity (logP = 1.56) and absence of esterase in VH, thus limiting degradation. The second hypothesis, however, is to be taken with caution. There is, in fact, esterase activity in VH [260]; and acetylcholinesterase, causing heroin to hydrolyze into 6-MAM and 6-MAM into morphine, is present in VH in many animal species [261]. In the absence of hard evidence, the possibility that this activity is merely weaker or more saturable in VH than in blood or other organs cannot be excluded. Another hypothesis holds that the properties of esterases in VH are different from those in blood, as demonstrated for brain synapse acetylcholinesterase, which was unable to hydrolyze heroin, unlike erythrocyte acetylcholinesterase [262].

When 6-MAM is undetected in blood, the morphine/codeine ratio in blood or urine is sometimes used to determine whether the detected morphine resulted from codeine metabolism (morphine/codeine ratio <1) or from direct intake of morphine, and thus possibly of heroin (morphine/codeine ratio >1) [263]. Lin et al. [264] reported that the morphine/codeine ratios in 223 opiate-positive VH samples were systematically >1 when 6-MAM was also detected, and moreover, were close to these found in blood. Rees et al. [259] also found that the VH morphine/codeine ratio was useful in revealing heroin intake. The low codeine concentrations found in VH, however, close to the quantification limit, may hinder the use of VH for this purpose.

#### Benzodiazepines

In a postmortem analysis of 3 nitro-benzodiazepines (nitrazepam, flunitrazepam and clonazepam) and their 7-amino metabolites, Robertson and Drummer [265] reported that in 15 % of cases in which 7-amino metabolites were detected in blood, they were not detected in VH. Moreover, the parent drugs were detected in VH in only 10 % of cases versus 30 % in blood. This differential positivity may have been due to the fact that VH benzodiazepine levels were generally one-third of those in blood. In a series of 17 postmortem cases, Scott and Oliver [266] assayed three benzodiazepines (diazepam, nordazepam and temazepam) in blood and in VH; in seven cases, one or more were detected in blood but not in VH. These results may reflect the fact that benzodiazepines are highly bound to proteins; their neutral or weak acidic properties further decrease diffusion into VH, which may be alkaline, as observed in postmortem samples, with a mean pH value of 8.3 and range of 7.3–9.1 [267].

#### Other compounds

VH also appears useful, in the absence of blood, for revealing use of cocaine [104, 268]. Moreover, the detection window is wider than in blood, as seen from the cases where cocaine is detected in VH but not in blood [91]. Jenkins and Oblock [269] showed that phencyclidine (PCP) was systematically detected in VH when detected in blood and/or urine. Cox et al. [270] confirmed this qualitative interest for PCP in a series of 26 autopsies. Oxycodone [271] and phenytoin [90] were systematically detected in VH when detected in blood in a respective series of 30 and 12 cases.

These studies confirm the usefulness of VH in detecting xenobiotic consumption. Moreover, for certain compounds (6-MAM, cocaine, and PCP), the VH detection window is wider than that for blood. For other compounds such as benzodiazepines, the qualitative importance of VH seems more limited. However, this may be related to analytic techniques: the same analysis protocols as in blood are usually applied in VH, despite the fact that concentrations are generally lower. Dedicated techniques developed and validated for VH could lower detection thresholds and increased detection rates. This may be difficult to achieve with present-day analytic techniques for some compounds with very low VH concentrations, however, such as tetrahydrocannabinol (THC) or its metabolites (11-OH-THC, THC-COOH or THC-COOH glucuronide) [272].

### Quantitative interpretation

#### Opiates

In 20 cases of death by heroin, Scott and Oliver [116] reported lower morphine concentrations in VH than in blood, with a significant correlation (*r*^2^ = 0.697) between the two. In light of this correlation, the authors considered VH to be the “ideal matrix” for analyzing morphine in the absence of blood. 6-MAM concentrations in VH were higher than in blood, but without correlation. Rees et al. [259] confirmed these findings, but pointed out that the correlation between VH and blood morphine concentrations depends on the intake-to-death interval, and may also be affected by intake modalities. The authors concluded that blood morphine concentration could not be extrapolated from the VH level. In the same study, it was shown that codeine concentrations were higher in VH than in blood, with a correlation with femoral blood level (*r*^2^ = 0.672). The authors suggested that codeine’s greater lipophilicity might account for higher VH concentrations than blood concentrations, unlike with morphine (logP codeine = 1.39; morphine = 0.87). Knittel et al. [271] reported that oxycodone showed a positive linear correlation between VH and blood concentrations, but with too great a scatter for extrapolation of levels from VH to blood.

#### Cocaine

More than in the case of other substances, postmortem blood concentrations of cocaine rarely correspond to those at time of death, largely due to strong in corpore and in vitro degradation. VH is one of the tissues in which xenobiotic composition is considered relatively stable over the early postmortem period, and its application as a matrix for cocaine quantification has naturally been widely studied. Results from several studies have, however, been divergent.

Antonides et al. [118], in a series of 40 autopsies, reported that VH cocaine concentrations were higher than in blood in 72 % of cases. This was confirmed by Logan and Stafford [98], who, moreover, found no correlation between the two matrices. The authors attributed these higher concentrations in VH to greater degradation of cocaine in blood. To circumvent the uncertainties of degradation, Duer et al. [119] investigated correlation for what they termed “total cocaine”, corresponding to the sum of the concentrations (in *µ*mol/L) of cocaine and its metabolites (ecgonine, ecgonine methyl ester and benzoylecgonine). In this condition, correlations of 0.939 and 0.883 were obtained between VH and femoral blood levels, and between VH and cardiac blood levels, respectively. Thus the authors concluded that VH was as reliable as blood for cocaine analysis. Fernandez et al. [104] reported VH cocaine concentrations near to those of blood (mean ratio, 1.03; range, 0.36–2.94), with a significant correlation coefficient (*r* = 0.71). According to the authors, VH could confirm the presence of cocaine in absence of blood but by no means could it estimate the blood concentration accurately. Carvahlo et al. [273] reported excellent correlation between VH and blood levels for cocaine (*r* = 0.98) and benzoylecgonine (*r* = 0.95) in 7 deaths by cocaine overdose but not in the 11 cases of accidental death. Another study showed that mean concentrations (*n* = 53) of cocaine and cocaethylene did not significantly differ between blood and VH, unlike benzoylecgonine, and reported correlations between blood and VH levels for benzoylecgonine (*r* = 0.763) and cocaine (*r* = 0.854), but not for cocaethylene (*r* = 0.343) [268].

These divergences highlight the importance of parameters that cannot be controlled in postmortem cases (intake-to-death time, time from death, postmortem redistribution and stability). Thus, blood cocaine level at death may not be extrapolated with precision from VH level alone.

#### Benzodiazepines

In 52 postmortem cases in which nitro-benzodiazepines (nitrazepam, flunitrazepam and clonazepam) and their 7-amino metabolites were assayed in blood and in VH, Robertson and Drummer [265] reported a correlation of *r* = 0.626 for the parent drugs and *r* = 0.764 for the metabolites. According to the authors, such reasonable positive correlations, also found for the metabolites in urine, bile and liver, enabled more precise interpretation of the blood data. In a series of 17 autopsies, Scott and Oliver [266] reported coefficients of determination (*r*^2^) of 0.788 for temazepam, 0.723 for diazepam and 0.068 for nordiazepam. In all cases, the VH levels were lower than in blood. Although correlations were identified, the authors reported a wide scatter in the results, probably related to variations in parameters such as intake modality, intake-to-death interval and time to autopsy. Finally, another autopsy study showed non-significant trends for nordazepam (*n* = 58, *r*^2^ = 0.473), bromazepam (*n* = 31, *r*^2^ = 0.345) and oxazepam (*n* = 28, *r*^2^ = 0.588) between VH and blood [274]. It thus seems that quantitative interpretation of VH benzodiazepine concentrations cannot consist in straightforward extrapolation of blood levels, given the weak correlations and the scatter found in the results.

#### Gamma-hydroxybutyric acid

Gamma-hydroxybutyric acid (GHB) is naturally present in organisms. Moreover, potentially significant postmortem neoformation of an unclear origin has been reported [275]. GHB was also used in anesthesiology, and is misused as a recreational drug and to incapacitate a victim. The main objective in the interpretation of postmortem blood concentration is to determine whether the origin was purely endogenous or involved exogenous administration. Postmortem neoformation misleads us into interpreting an elevated blood level as indicative of an exogenous origin. VH is one of the alternative matrices proposed to confirm elevation in cardiac blood level [276, 277]. Kintz et al. [277] described an interpretation tree for determining exogenous origin, with a 50 mg/L threshold in cardiac blood, and confirmed, when positive, by the same threshold in femoral blood and VH. Moriya and Hashimoto [278] suggested a 10 mg/L threshold in urine and VH. Another study, in which cardiac and femoral blood, VH, urine and cerebrospinal fluid was analyzed, found that VH levels could exceed blood levels, and indeed, were sometimes the highest of any of the five matrices [275]. VH concentrations were systematically below 50 mg/mL but were often greater than 10 mg/mL. For interpretation of blood GHB, the authors concluded that VH should not be the sole alternative matrix. In a recent review, Castro et al. [279] stressed that the thresholds should be seen as interpretation aids on a case-by-case basis rather than as hard facts. Moreover, the authors observed that the thresholds for GHB reported in the various matrices showed a tendency to become lower with increasing expertise in sampling and storage.

#### Insulin

Insulin determination in postmortem blood is complex, especially in hemolyzed specimens, due to matrix interference and insulin degradation by insulin-degrading enzymes and a non-proteolytic process initiated by hemoglobin [280]. Thus, interpretation of blood insulin concentrations in postmortem investigation is often tricky. The determination of insulin in VH appears to hold promise, due to low analytical background, less pronounced postmortem changes [190] and the absence of hemoglobin. In four studies of populations with no history of diabetes or in subjects with type 1 diabetes where the cause of death was unrelated to insulin overdose, insulin was either undetectable in VH or shown in concentrations close to the limit of detection. These studies used various analytical methods: LC/MS–MS, limit of detection (LOD) = 2.4–4.8 µIU/mL, *n* = 10 [190]; LC/MS–MS, LOD = 4.5 µIU/mL, *n* = 46 [192]; chemiluminescence enzyme immunoassay, LOD = 0.2 µIU/mL, *n* = 40 [191]. The fourth study, by Nowicka et al. [193], was a comment article which reported insulin determination by immunoradiometric assay on 93 VH samples from a random autopsy population: insulin was not detected in 86 cases (LOD = 0.5 µIU/mL) and ranged from 1.42 to 24.42 µIU/mL in the seven remaining cases (the outlier with 24.42 µIU/mL was probably related to insulin administration, as the subject was not known to be diabetic, and an insulin syringe was found near the corpse). In some reported cases of death related to insulin overdose in which VH analysis has been used (cf. Table 2), VH insulin concentrations were notably higher (from 11.5 to 103 µIU/mL: five cases). These studies demonstrated that VH is an interesting matrix to sample and analyze as a complement to blood or serum in postmortem investigation of insulin intoxication. However, although Ojanperä et al. [281] demonstrated success in the detection of insulin or metabolites by HPLC coupled with high-resolution mass spectrometry in three cases of non-insulin-related death of diabetes mellitus subjects with postmortem intervals between 4 and 8 days, more data are needed on insulin stability over postmortem intervals.

#### MDMA

In a rabbit model, De Letter et al. [89] demonstrated a correlation between MDMA concentrations in VH and blood after concentrations reached equilibrium (i.e., about 1 h after administration). In this study, VH concentrations were more stable than those in blood in the case of long postmortem time (73 h), and thus more representative of antemortem blood levels.

#### Other compounds

Jenkins and Oblock [269] and Cox et al. [270] found no correlation between blood and VH PCP levels in 30 and 26 cases, respectively. Holmgren et al. [73] studied correlations between blood and VH concentrations in 46 compounds of various groups of drugs in samples stored with KF at −20 °C for 12 months. Correlations emerged for a half of the substances (*n* = 23), including amphetamine, diltiazem, tramadol and venlafaxine, while for compounds such as clomipramine, clozapine and sertraline, no correlation was observed. Given the lack of discussion of these results and the small sample sizes for certain drugs (e.g., tramadol: *n* = 4), these findings do not warrant extrapolation from VH to blood, but may serve as a basis for future studies.

As described above, the main approach used for the extrapolation of blood levels at death from VH levels is based on correlations. However, Bévalot et al. [88] proposed the use of statistical data processing for interpreting meprobamate concentrations in VH, determining a VH concentration threshold to distinguish therapeutic from overdose levels. In a 117-case series (40 deemed therapeutic, 77 overdose), a threshold of 28 mg/L was determined for VH meprobamate, with sensitivity of 0.95 and absolute specificity of 1. In the same case series, an interpretation table was described for the probability of an association between a given VH level and a blood level in one of four concentration ranges: <30, 30–50, 50–100 and >100 mg/L. Using a similar approach, Parker and McIntyre [282] reported that VH quetiapine concentrations in non-toxic deaths (*n* = 8) ranged between 0.10 and 0.22 mg/L (95 % confidence interval [CI]), and in toxic deaths (*n* = 8) between 0.74 and 1.74 mg/L (95 % CI).

## Survival time

Several authors have proposed the use of blood/VH concentration ratios for estimating survival time (intake-to-death interval) based on the time of distribution from blood to VH: soon after intake, the ratio is higher than when equilibrium between the two matrices is reached. Using inquest data providing an estimate of last intake time, Scott and Oliver [266] showed that the blood/VH concentration ratios for benzodiazepines were higher in rapid death. However, the authors stressed that data were lacking for various postmortem factors notably redistributions that were liable to impact the ratio. Teixeira et al. [283] demonstrated in a rabbit model that the blood/VH concentration ratio after intramuscular administration of diazepam was 20 up to 1 h, and then fell to 4.5 by 6 h. At equilibrium, 1–2 h post-administration, the ratio was 10. The authors suggested the blood/VH concentration ratio as a “complementary tool” for determining intake-to-death time, without giving further details of how it could be used. Antonides et al. [118], also investigating circumstances of death, reported that when blood concentrations of cocaine were higher than in VH, death had occurred sooner after intake. In these cases, blood benzoylecgonine levels were up to twofold higher than VH levels.

## Postmortem redistribution

De Letter et al. [56] reported that the postmortem distribution of MDMA in a rabbit model showed VH concentrations to be more stable, and representative of antemortem rather than postmortem blood levels. VH MDMA levels, however, were especially elevated in the wall of the eyeball, so diffusion was a possibility, especially in the case of long postmortem time. The findings of a previous study of 73-h postmortem evolution of VH MDMA concentrations suggested that such accumulation contributed only moderately to VH concentration [89]. For digoxin, a digitalis derivative, Ritz et al. [92] reported very high concentrations in choroid and retinal tissue (63.9–485 ng/g), close to levels found in cardiac muscle and higher than those in VH (2.2–7.1 ng/mL), based on the results of an autopsy series (*n* = 19). A similar distribution was found for digitoxin [284]. The authors suggested that these differences could induce postmortem redistribution from choroid and retinal tissue to VH. In a study of postmortem redistribution of cocaine in a pig model, McKinney et al. [285] sacrificed the animals 5 min after intravenous administration, and performed sampling at sacrifice and 8 h later. While blood concentration had not changed, VH levels had risen considerably. The authors expected such a rise caused by redistribution from periorbital blood, but not to that degree: baseline concentrations were significantly lower in VH (mean = 939 ng/mL) than in blood (mean = 3245 ng/mL), whereas by 8 h, the two were close (VH, mean = 3067 ng/mL; blood, mean = 3568 ng/mL). They hypothesized that intraocular tissue such as the retina might be a region of accumulation, with postmortem release to VH. Teixeira et al. [283] found a twofold elevation of VH diazepam concentrations and a threefold elevation of nordazepam during a 24 h postmortem period in an animal model. Maskell et al. [286] investigated the postmortem redistribution of the heroin metabolites morphine and morphine-3-glucuronide (M3G) in nine biological matrices in a rabbit model. In VH collected at 24 h postmortem, a 181 % increase in free morphine concentration and a 425 % increase in total morphine concentration were observed. For M3G, the increase in concentration was 1.002 %, and among the nine matrices studied, VH was the only one in which the M3G concentration increased. The authors explained the increase in morphine and M3G concentrations in VH by diffusion from “tissue” without, however, specifying the tissue in question.

These studies suggest that VH is a matrix protected against the main sources of postmortem redistribution from the abdominal cavity, but that ocular tissue may be a region of accumulation of xenobiotics liable to diffuse into VH postmortem.

## Conclusions

When blood is lacking or is modified by postmortem factors, alternative matrices may be useful. An ideal alternative should enable detection of the same xenobiotics as found in blood, at correlated concentrations, and without the postmortem effects. VH is the matrix that probably comes closest to this ideal. Moreover, from a practical point of view, VH is easy to sample, and compounds within it tend to be stable if certain storage conditions are warranted and analysis is straightforward, with no more preparation required than is necessary to ensure “cleanliness”. It is of particular screening interest in the absence of blood, as most compounds of forensic interest are detected from VH. For several compounds (6-MAM, PCP, cocaine), moreover, the detection window is wider than that in blood. This qualitative interest could be enhanced by dedicated techniques achieving lower detection thresholds than those of most other complex forensic matrices.

The limitations of VH for the purpose of forensic toxicology largely concern quantitative interpretation. Various controlled animal or autopsy studies have been conducted to determine the implication of a xenobiotic in a victim’s death by interpreting only VH concentrations. Their findings show that VH and blood concentrations do not correlate for all compounds, and that in others, the scatter of the autopsy data usually precludes extrapolation to blood concentrations without significant error. This scatter reflects various non-controllable and often unknown parameters such as survival time, postmortem time, ophthalmic pathology and drug interaction. To optimize quantitative interpretation, various possibilities must be considered, the first of which is improving our knowledge of xenobiotic distribution in VH. Although diffusion seems to be the preponderant mechanism taking place for most compounds, diffusion from blood is not merely passive, and an enhanced understanding is needed of the role and mechanisms of active transport in antemortem distribution of compounds of forensic interest. Second, it is important to explore distribution in ocular tissues, and particularly in the choroid and retina, which may act as accumulation regions, with possible postmortem redistribution toward VH. Third, statistical tools must be developed and implemented in order to assess the uncertainty of interpretation of VH concentrations to the greatest degree possible. Even more than for concentrations in blood, it is important to report and discuss the uncertainty of findings obtained from alternative matrices according to the specific data for forensic cases.
